# Fangchinoline Inhibits Human Esophageal Cancer by Transactivating ATF4 to Trigger Both Noxa-Dependent Intrinsic and DR5-Dependent Extrinsic Apoptosis

**DOI:** 10.3389/fonc.2021.666549

**Published:** 2021-06-14

**Authors:** Yunjing Zhang, Shiwen Wang, Yukun Chen, Junqian Zhang, Jing Yang, Jingrong Xian, Lihui Li, Hu Zhao, Robert M. Hoffman, Yanmei Zhang, Lijun Jia

**Affiliations:** ^1^ Cancer Institute, Longhua Hospital, Shanghai University of Traditional Chinese Medicine, Shanghai, China; ^2^ Department of Laboratory Medicine, Huadong Hospital Affiliated to Fudan University, Shanghai, China; ^3^ Department of Surgery, University of California, San Diego, San Diego, CA, United States; ^4^ Anticancer Inc., San Diego, CA, United States

**Keywords:** fangchinoline (FCL), esophageal squamous cell carcinoma (ESCC), cell cycle, intrinsic apoptosis, extrinsic apoptosis

## Abstract

Esophageal squamous cell carcinoma (ESCC) is a recalcitrant cancer. The Chinese herbal monomer fangchinoline (FCL) has been reported to have anti-tumor activity in several human cancer cell types. However, the therapeutic efficacy and underlying mechanism on ESCC remain to be elucidated. In the present study, for the first time, we demonstrated that FCL significantly suppressed the growth of ESCC both *in vitro* and *in vivo*. Mechanistic studies revealed that FCL-induced G1 phase cell-cycle arrest in ESCC which is dependent on p21 and p27. Moreover, we found that FCL coordinatively triggered Noxa-dependent intrinsic apoptosis and DR5-dependent extrinsic apoptosis by transactivating ATF4, which is a novel mechanism. Our findings elucidated the tumor-suppressive efficacy and mechanisms of FCL and demonstrated FCL is a potential anti-ESCC agent.

## Introduction

Esophageal squamous cell carcinoma (ESCC) is the major histologic subtype of esophageal cancer, and its incidence and fatality keep rising at an alarming rate worldwide (1). Despite the considerable progress in diagnosis and treatment of ESCC, the present therapeutic strategies, including chemotherapy, radiation and surgery, still have high recurrence and metastasis rates (2). Moreover, the developments of therapeutic targets and targeted drugs remain ineffective (3). Therefore, safe and effective therapeutic approaches for ESCC are urgently needed.

**Figure 1:**
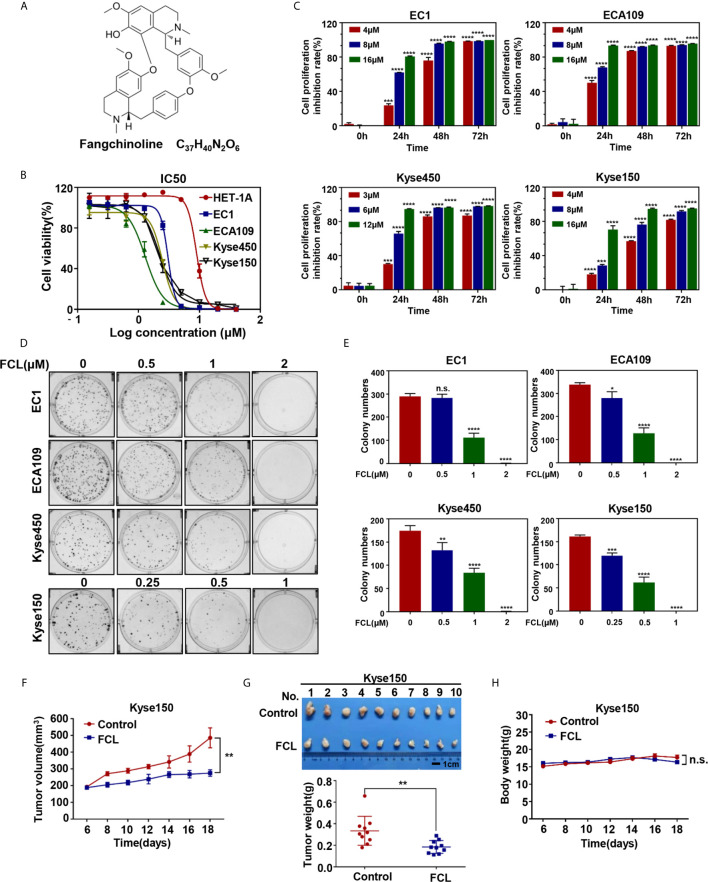
Efficacy of Fangchinoline on ESCC *in vitro* and *in vivo*. **(A)** Chemical structure of FCL. **(B)** Human esophageal epithelial cell line HET-1A and ESCC cell lines EC1, ECA109, Kyse450, Kyse150 were treated with indicated concentrations of FCL for 72 hours, and cell viability was determined by ATPlite assay. Representative inhibitory curves for each cell line are shown. **(C)** ATPlite assay was used to determine the cell growth of different ESCC cell lines at the indicated concentrations of FCL for 0, 24, 48 and 72 hours. **(D)** Representative images of three independent experiments are shown for the inhibition of colony formation by FCL. **(E)** Graph of the relative number of colonies formed. **(F)** Nude mice were subcutaneously transplanted Kyse150 cells and treated with FCL as indicated in Materials and Methods. Tumor size was determined with caliper every other day, and the volume was calculated to construct a growth curve. **(G)** Mice were sacrificed, and tumor tissues were harvested and photographed. The tumor weight was measured with an electronic scale on the sacrificed day. **(H)** Mouse body weight was recorded every other day during the whole experiment. *denotes *P* < 0.05, **denotes *P* < 0.01, ***denotes *P* < 0.001, ****denotes *P* < 0.0001, n.s. denotes not significant.

Currently, Chinese herbal medicinal agents have made great progress in the treatment of human cancers due to the relatively high efficacy and few side effects (4). The Chinese herbal monomer fangchinoline (FCL), extracted from the traditional Chinese herbal alkaloid tetrandrine root, characterizing as a new compound sharing structural features with tetrandrine (**Figure 1A**) (5). FCL has been shown to have a wide range of pharmacological activities such as anti-inflammation, anti-oxidation and anti-thrombosis activities (6–9). Remarkably, FCL exerts substantial anti-tumor efficacy on many types of human tumor cells by arresting cell cycle, inhibiting metastasis, as well as triggering apoptosis (10–12). For example, it was reported that FCL inhibited cell growth in lung cancer cells and melanoma cells by targeting the FAK pathway (13, 14). Furthermore, FCL induced apoptosis of breast cancer cells and glioblastoma cells by activating the PI3K/Akt/GSK-3β pathway (15, 16). However, the anti-tumor efficacy of FCL on ESCC and it's underlying mechanism has not been investigated.

In the present study, for the first time, we reported that FCL effectively suppressed the tumor progression of ESCC by triggering cell-cycle arrest and apoptosis. More importantly, we reported a novel mechanism by which FCL transactivated ATF4 to trigger both Noxa-dependent intrinsic and DR5-dependent extrinsic apoptosis. Our study revealed the tumor suppressive efficacy of FCL on ESCC, and validated FCL as a potential anti-ESCC agent.

## Materials and Methods

### Reagents

Fangchinoline was purchased from MCE (MedChem Express, Shanghai, China), and the purity of the compounds was ≥99.92%. FCL was dissolved in dimethyl sulfoxide (DMSO) and stored at −80°C for the *in vitro* study. For the *in vivo* study, FCL was dissolved first in 5% DMSO and then in 10% 2-hydroxypropyl-β-cyclodextrin (Sangon Biotech, Shanghai, China).

### Cell Culture

Human esophageal epithelial cell line HET-1A and human ESCC cell lines EC1, ECA109, Kyse450, Kyse150 were obtained from the Type Culture Collection of the Chinese Academy of Sciences (Shanghai, China) and cultured in Dulbecco’s Modifed Eagle’s Medium (DMEM, hyclone, Logan, UT), containing 10% fetal bovine serum (FBS, Biochrom AG, Berlin, Germany) and 1% penicillin–streptomycin solution (Gibco, USA) at 37°C with 5% CO_2_.

### Cell Viability and Clonogenic Survival Assay

Cells were seeded in black 96-well plates with 2×10^3^ cells per well in triplicate and cultured overnight. Cells were treated with DMSO or FCL at the indicated concentrations for 0, 24, 48 and 72 hours. At the end of the incubation, the cell viability was measured by ATPlite luminescence assay (PerkinElmer, Norwalk, CT, USA) according to the manufacturer’s protocol. For clonogenic survival assay, cells were plated into six-well plates (300 cells per well) in triplicate and allowed to adhere overnight. Cells were treated with the indicated concentrations of FCL and cultured for 12 days. Cells were stained with crystal violet and the colony number was counted. Colonies with more than 50 cells each were counted and photographed with a gel imager (GelDoc XR System, Bio-rad, USA).

### Cell Cycle Analysis

For cell cycle analysis, cells were treated at the indicated concentrations of FCL for 24 hours. FCL-treated cells or control cells were harvested and fixed in 70% ethanol at -20°C overnight. Then, the fixed cells were stained with propidium iodide (PI, 36 μg/mL; Sigma, St. Louis, MO, USA) at 37°C for 15 min, and performed for fluorescence activated cell sorting (FACS) analysis by Flow Cytometry (BD FACSVerse™, New Jersey, USA). Data were analyzed with FlowJo 7.6 software.

### Apoptosis Assay

For apoptosis analysis, cells were treated at the indicated concentrations of FCL for 24 hours. FCL-treated cells or control cells were collected and washed with cold PBS, and then stained with an AnnexinV-FITC and PI Apoptosis Kit according to manufacturer’s instructions (Yuheng Biotechnology, Suzhou, China). Apoptotic cells were analyzed by Flow Cytometry (BD FACSVerse™, New Jersey, USA). Data were analyzed with FlowJo 7.6 software.

### Western Blot Analysis

Total protein was collected using RIPA (Radio Immunoprecipitation Assay) lysis buffer and resolved by 7.5-15% SDS-PAGE, followed by transferring the proteins to an Immobilon-PVDF Membrane (Merck Millipore Ltd, Tullagreen, lreland). The membrane was then blocked with 5% skim milk for 1 hour followed by incubation with the primary antibodies overnight as follows, cleaved caspase-8 (c-CASP8), ATF4, CHOP, DR5, Noxa, p27, Bax, Bid (Cell Signaling Technology, Danvers, MA, USA), cleaved caspase-3 (c-CASP3), cleaved caspase-9 (c-CASP9), cleaved PARP(c-PARP), PARP, β-actin (HuaBio, China), p21(Proteintech, Chicago, USA), CyclinE, CDK2, CDK4, CDK6, Fas, DR3 (Santa Cruz Biotechnology, Santa Cruz, CA, USA). Corresponding second antibodies were incubated for 1 hour and membranes photographed by Tanon 5200 visualizer (Shanghai, China).

### RNA Extraction and Real-Time PCR

Total RNA was isolated using the Ultrapure RNA Kit (Cwbiotech, Beijing, China) according to the manufacturer’s instructions. The reverse transcription reaction was performed on 1 μg of total RNA per sample using the PrimerScript reverse transcription reagent kit (TaKaRa, Shiga, Japan) according to the manufacturer’s instructions. After reverse transcription, the real-time polymerase chain reaction (PCR) was performed using the Power SYBR Green PCR MasterMix (Applied Biosystems, Foster City, CA) on the ABI 7500 thermocycler (Applied Biosystems) following the instrument instructions. For each sample, the mRNA abundance was normalized to the amount of β-actin. The sequences of the primers were as follows:

for β-actin, forward: 5′-CGTGCGTGACATTAAGGAGAAG-3′,
reverse: 5′-AAGGAAGGCTGGAAGAGTGC-3′;
for ATF4, forward: 5′-ATGACCGAAATGAGCTTCCTG-3′,
reverse: 5′-GCTGGAGAACCCATGAGGT-3′;
for DR5, forward: 5′-CCAGCAAATGAAGGTGATCC-3′,
reverse: 5′-GCACCAAGTCTGCAAAGTCA-3′;
for Noxa, forward: 5′-ACCAAGCCGGATTTGCGATT-3′,
reverse: 5′-ACTTGCACTTGTTCCTCGTGG-3′.

### siRNA Silencing

The cells were transfected with siRNA oligonucleotides against the following genes using the Lipofectamine RNAiMAX Transfection Reagent (Invitrogen, USA), according to the manufacturer’s instructions. The sequences of siRNA were as follows:

siControl: 5′-UUCUCCGAACGUGUCACGUTT-3′;
siATF4-1: 5′-CCAAAUAGGAGCCUCCCAUTT-3′;
siATF4-2: 5′-CCTCACTGGCGAGTGTAAA-3′;
siDR5: 5′-AAGACCCUUGUGCUCGUUGUC-3′;
siNoxa: 5′-GGUGCACGUUUCAUCAAUUUGTT-3′;
sip21: 5′-GACCAUGUGGACC UGUCAC-3′;
sip27: 5′-CCGACGATTCTTCTACTCA-3′.

### Subcutaneous Transplantation Tumor Model

BALB/c nude female mice were purchased from Lingchang Biological Technology Co., Ltd. (Shanghai, China). All mice were kept and bred in a specific pathogen-free environment in the animal facility of Longhua hospital. The mice were maintained in a temperature−controlled room (22 ± 2°C) with a 12−hours light/12−hours dark cycle and a relative humidity of 40−60%, and were given free access to sterilized food and water. Animal experiments were performed in accordance with the National Guidelines for Experimental Animal Welfare, with approval from the Institutional Animal Care and Use Committee of Longhua hospital, Shanghai University of Traditional Chinese Medicine.

Briefly, 4×10^6^ Kyse150 cells were subcutaneously injected into the bilateral flank of each mouse, and mice were randomly assigned to control and FCL-treatment groups (five mice per group). Each mouse was treated with either β-cyclodextrin crystalline (vehicle control) or FCL (100 mg/kg) *via* intraperitoneal injection once a day for 13 consecutive days. The day of tumor appearance was designated day 1 (6 days after xenografting). Tumor size was measured with a caliper and tumor volume was calculated using ellipsoid volume formula (length×width2)/2. The body weights of the mice were measured with an electronic scale every other day. Tumor tissues were harvested, photographed, and weighed at the end of the experiment.

### Statistical Analysis

The statistical significance of differences between groups was assessed using GraphPad Prism7 software (GraphPad Software, Inc., San Diego, CA, USA). All data were presented as mean ± Standard Error of Mean. The student’s t-test was used for the comparison of parameters between two groups. P-value of *P* < 0.05 was significant, n.s.=not significant. For all tests, four levels of significance (*P < 0.05, **P < 0.01, ***P < 0.001, ****P < 0.0001) were used.

## Results

### Fangchinoline Suppressed the Tumor Growth of ESCC *In Vitro* and *In Vivo*

We first evaluated the efficacy of FCL on normal human esophageal epithelial cell and ESCC cells. Our results showed that the IC50 values of FCL for the normal human esophageal epithelial cell line HET-1A and ESCC cell lines EC1, ECA109, Kyse450, Kyse150 were 8.93, 3.042, 1.294, 2.471 and 2.22 μM, respectively (**Figure 1B**). Furthermore, we found a time and dose-dependent growth inhibition in the four ESCC cell lines (**Figure 1C**). FCL inhibited colony formation of ESCC cells in a dose-dependent manner (**Figures 1D, E** **)**. These findings indicated that FCL suppressed the viability of ESCC cells. To further assess the efficacy of FCL, we established a subcutaneous-transplantation tumor model of human esophageal cancer in mice by using Kyse150 cells. As shown, FCL significantly inhibited tumor growth over time compared with the control group (*P* < 0.01, **Figure 1F**). Notably, FCL-treated group mice developed smaller tumors than the control group by tumor weight analysis (*P* < 0.01, **Figure 1G**). During the whole experiment, there was no substantial change in the body weights of mice between the control group and FCL treatment group, suggesting no general toxicity of FCL treatment (**Figure 1H**). Collectively, our findings indicated that FCL inhibited the tumor growth of ESCC both *in vitro* and *in vivo*.

### Fangchinoline Induced G1-Phase Cell-Cycle Arrest of ESCC Cells

**Figure 2:**
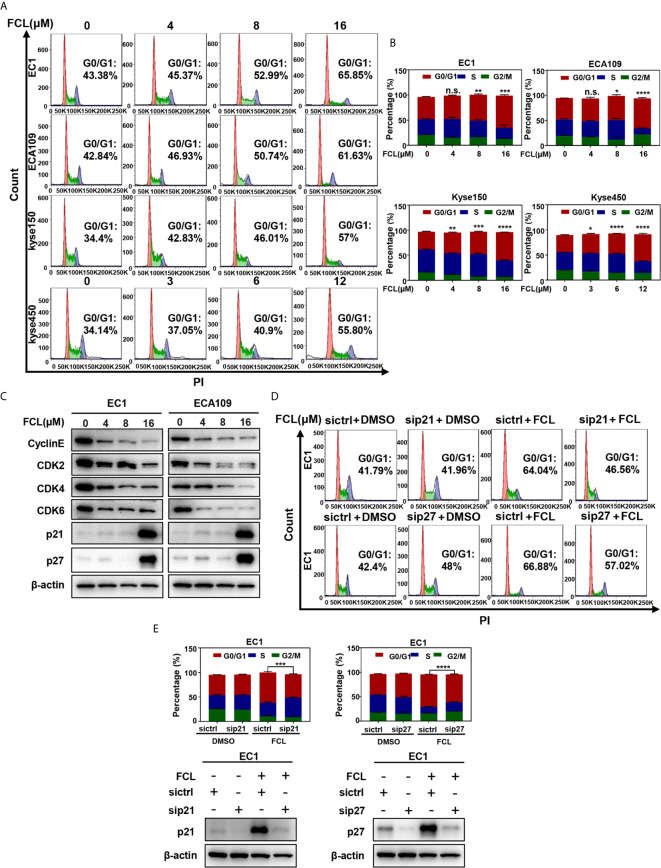
Fangchinoline arrested ESCC cells in G1 phase. **(A, B)** ESCC cells were pre-incubated with DMSO or FCL for 24 hours, followed by PI staining and FACS analysis for cell‐cycle profiling. **(C)** FCL‐induced decrease of CyclinE, CDK2, CDK4, CDK6 was accompanied by the accumulation in p21 and p27. After 24 hours of FCL treatment at the indicated concentrations, EC1 and ECA109 cells were subjected to Western blotting using antibodies against Cyclin E, CDK2, CDK4, CDK6, p21 and p27 with β-actin as a loading control. **(D)** EC1 and ECA109 cells were transfected with control or p21 or p27 siRNA (72 hours), treated with 16 μmol/L FCL (24 hours), and subjected to PI staining and FACS analysis. **(E)** The percentage of cells at the G0/G1 phase was indicated. The protein levels of p21 or p27 were determined by Western blotting analysis with β-actin as a loading control. *denotes *P* < 0.05, **denotes *P* < 0.01, ***denotes *P* < 0.001, ****denotes *P* < 0.0001, n.s. denotes not significant.

To further explore the inhibitory mechanism of FCL on the viability of ESCC cells, the effect of FCL on cell cycle was determined. We found that cell populations in G0/G1 phase of cell cycle were significantly increased in EC1, ECA109, Kyse150 and Kyse450 cells in a dose-dependent manner (**Figures 2A, B** **)**. Owing to CyclinE and Cyclin-dependent kinases 2, 4 and 6 (CDK2/4/6) are key regulators in the G1 phase, we next determined the expression levels of indicated regulators in FCL-treated ESCC cells (17). Our data showed that FCL treatment obviously dropped the protein levels of CyclinE and CDK2/4/6 in both EC1 and ECA109 cells (**Figure 2C**), suggesting that FCL prevented G1 to S phase progression of ESCC cells.

In addition, we found that the cell cycle inhibitors p21 and p27, which inhibit CDK/Cyclin complexes (18, 19), were significantly accumulated upon FCL treatment in EC1 and ECA109 cells (**Figure 2C**). To further define the role of p21 and p27 in FCL-induced cell-cycle arrest, the expression of p21 or p27 was downregulated by siRNA silencing in FCL-treated EC1 cells. As shown in **Figures 2D, E**, p21 or p27 knockdown by siRNA significantly rescued the EC1 cells from FCL-induced G1 phase arrest. Taken together, our findings demonstrated that p21 and p27 played a crucial role in controlling G1 phase cell-cycle arrest elicited by FCL.

### Fangchinoline Triggered Apoptosis in ESCC Cells

**Figure 3:**
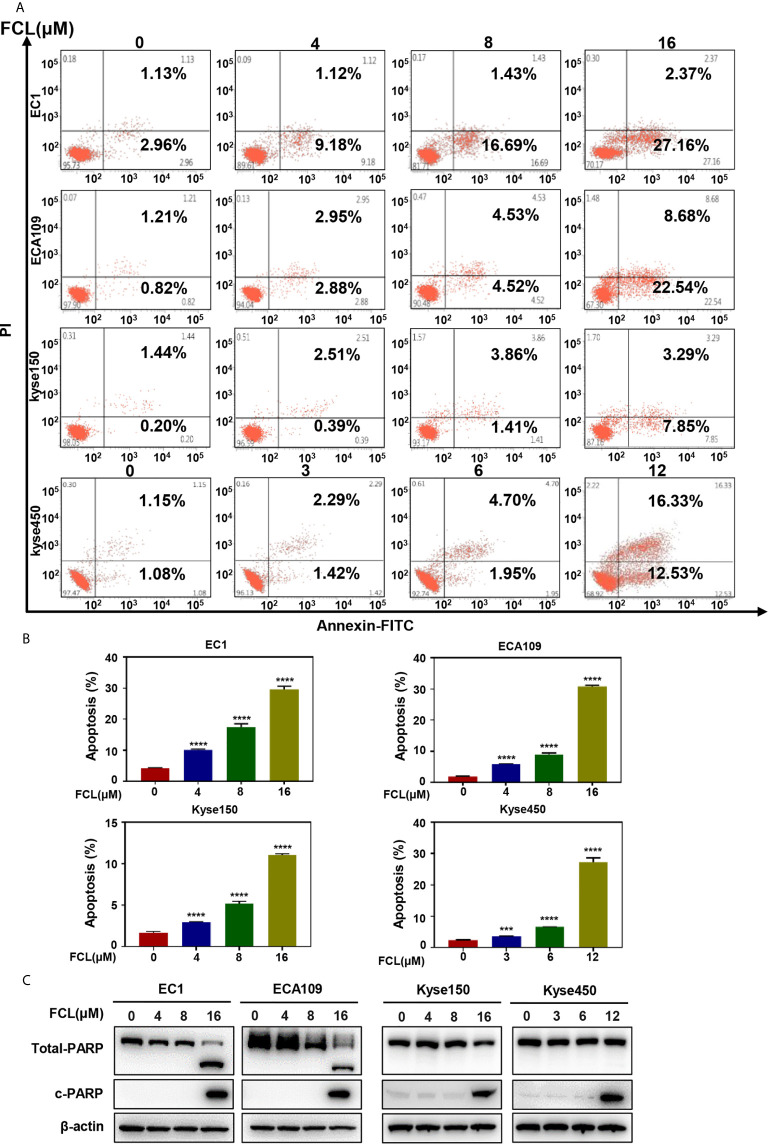
Fangchinoline induced apoptosis in ESCC cells. **(A, B)** ESCC cells were pre-incubated with the indicated concentrations of FCL or DMSO for 24 hours, and then the cells were detected with an annexin-V-FITC apoptosis detection kit and analyzed with FCAS. **(C)** FCL increased the proteins level of c-PARP. ESCC cells were treated at the indicated concentrations of FCL or DMSO for 24 hours, and cell lysates were analyzed by Western blotting with an antibody against c-PARP. ***denotes *P* < 0.001, ****denotes *P* < 0.0001.

After revealing that FCL disturbed the ESCC cells in G1 phase, we next examined the cellular responses to FCL treatment. We observed that FCL-treated ESCC cells presented the notable feature of apoptosis-shrunk morphology (**Supplementary Figure 1A**). PI and Annexin-V-FITC staining analysis confirmed that the number of Annexin V-positive cells (apoptosis marker) increased significantly after FCL treatment (**Figures 3A, B** **)**. Furthermore, FCL-treated ESCC cells had increased levels of cleaved PARP, a classical marker of apoptosis (**Figure 3C**). Therefore, our findings demonstrated that FCL triggered apoptosis in ESCC cells.

### Fangchinoline-Induced Intrinsic Apoptosis Mediated by Noxa

**Figure 4:**
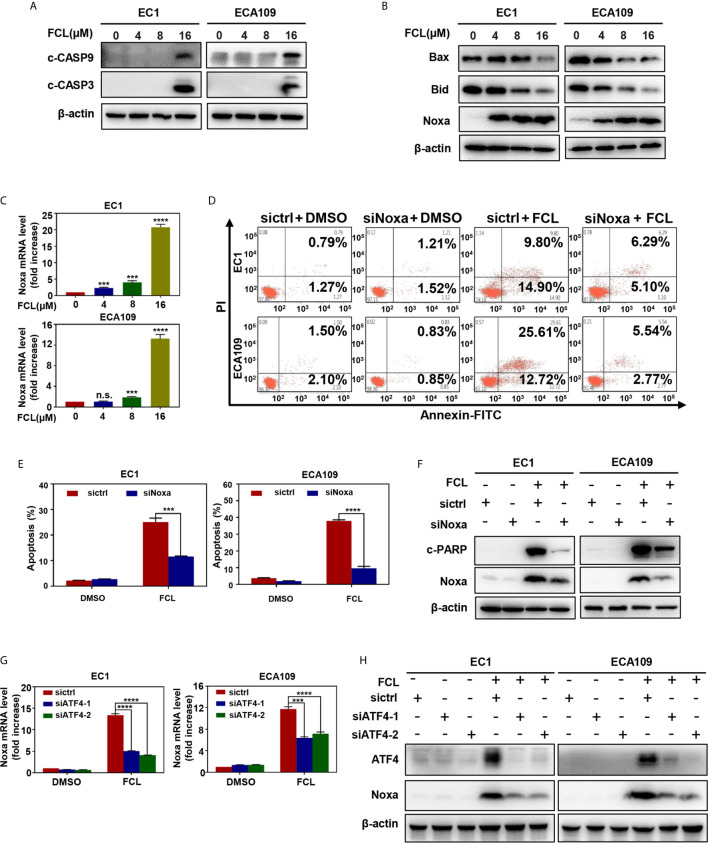
Fangchinoline triggered intrinsic apoptosis *via* the up‐regulation of Noxa. **(A)** FCL induced the activation of CASP9. EC1 and ECA109 cells were treated with FCL as described above and were subjected to Western blotting using antibodies against c‐CASP9 and c‐CASP3 with β-actin as a loading control. **(B)** The expression of classical pro‐apoptotic proteins Noxa, Bax and Bid were determined after FCL treatment. EC1 and ECA109 cells were treated with FCL at the indicated concentrations for 24 hours, followed by Western blotting using the indicated antibodies with β-actin as a loading control. **(C)** FCL increased the mRNA level of Noxa. The mRNA level of Noxa was determined by real-time PCR in EC1 and ECA109 cells. **(D, E)** Knockdown of Noxa inhibited apoptosis induced by FCL. EC1 and ECA109 cells were transfected with control or Noxa siRNA (72 hours), treated with FCL (16 μmol/L) for 24 hours. Apoptosis induction was quantified by Annexin V–FITC/PI double-staining analysis. **(F)** Apoptosis induction was quantified by Western blotting using an antibody against c‐PARP with β-actin as a loading control. **(G, H)** ATF4 is response for FCL-induced Noxa upregulation. EC1 and ECA109 cells were transfected (72 hours) with control or ATF4 siRNA, treated with FCL (16 μmol/L) for 24 hours. Expression of ATF4 and Noxa were assessed by Western blotting analysis. The effect of ATF4 on Noxa transcription was analyzed by real-time PCR. ***denotes *P* < 0.001, ****denotes *P* < 0.0001, n.s. denotes not significant.

To further characterize the mechanism underlying apoptosis in FCL-treated ESCC cells, we determined the expression of cleaved CASP9, a marker of intrinsic apoptosis. As shown in **Figure 4A**, FCL induced obvious accumulation of cleaved CASP9, as well as the upregulation of classical apoptotic hallmark cleaved CASP3 in EC1 and ECA109 cells, indicating that intrinsic apoptosis of ESCC cells was triggered by FCL. To explore the mechanism for activation of intrinsic apoptosis upon FCL treatment, we determined the expression of classical proapoptotic protein (Noxa, Bax and Bid). Strikingly, Noxa expression was obviously increased in both EC1 and ECA109 cells while Bax and Bid were downregulated (**Figure 4B**). Mechanistic studies showed that Noxa was transactivated by FCL (**Figure 4C**).

To further determine the potential role of Noxa in FCL-induced intrinsic apoptosis, the expression of Noxa was downregulated *via* siRNA silencing. Our data showed that Noxa knockdown with siRNA significantly suppressed FCL-induced intrinsic apoptosis, as evidenced by (i) the attenuated percentage of Annexin V-positive cells (**Figures 4D, E** **)**, and (ii) the reduction of the cleaved fragments of PARP (**Figure 4F**), demonstrating that FCL induced Noxa-dependent intrinsic apoptosis in ESCC cells. Given that Noxa could be transactivated by ATF4 (20, 21), we, therefore tested the potential involvement of ATF4 in FCL-induced Noxa expression in ESCC cells. As shown in **Figures 4G, H**, downregulation of ATF4 significantly inhibited the induction of Noxa at both mRNA (**Figure 4G**) and protein levels (**Figure 4H**) in EC1 and ECA109 cells, indicating that ATF4 transactivated Noxa upon FCL treatment.

### Fangchinoline Activated Extrinsic Apoptosis *via* the ATF4-DR5 Axis

**Figure 5:**
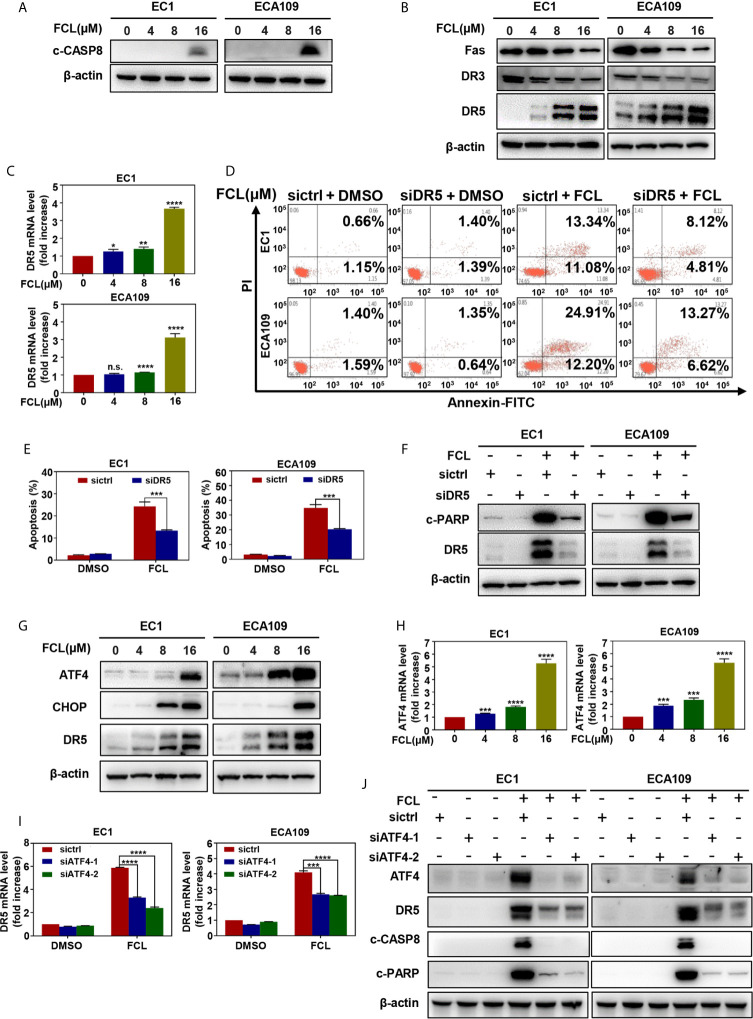
Fangchinoline activated extrinsic apoptosis *via* the ATF4-DR5 axis. **(A)** FCL induced the activation of CASP8. EC1 and ECA109 cells were treated with FCL as described above and were subjected to Western blotting using the antibodies against c‐CASP8 and c‐CASP3 with β-actin as a loading control. **(B)** The expression of death receptors Fas, DR3 and DR5 was determined. EC1 and ECA109 cells treated with FCL at the indicated concentrations for 24 hours, followed by Western blotting using the indicated antibodies with β-actin as a loading control. **(C)** FCL increased the mRNA level of DR5. The mRNA level of DR5 was determined by the real-time PCR in EC1 and ECA109 cells. **(D, E)** Knockdown of DR5 inhibited apoptosis induced by FCL. EC1 and ECA109 cells were transfected with control or DR5 siRNA (72 hours), and then treated with FCL (16 μmol/L) for 24 hours. Apoptosis induction was quantified by Annexin V–FITC/PI double-staining analysis. **(F)** Apoptosis induction was quantified by Western blotting using an antibody against c‐PARP with β-actin as a loading control. **(G)** FCL induced the accumulation of ATF4, CHOP and DR5. EC1 and ECA109 cells were treated with FCL at the indicated concentrations for 24 hours, followed by Western blotting using antibodies against ATF4, CHOP and DR5 with β-actin as a loading control. **(H)** The mRNA level of ATF4 was determined by real-time PCR in EC1 and ECA109 cells. **(I, J)** The expression of ATF4 mediated FCL-induced apoptosis in ESCC cells *via* ATF4-DR5 axis. ATF4 mediated FCL-induced DR5 upregulation. EC1 and ECA109 cells were transfected (72 hours) with control or ATF4 siRNA, treated with FCL (16 μmol/L) for 24 hours. Expression of ATF4, DR5, c-CASP8 and c-PARP was assessed by Western blotting. Transcriptional regulation of ATF4 on DR5 was analyzed by real-time PCR. *denotes *P* < 0.05, **denotes *P* < 0.01, ***denotes *P* < 0.001, **** denotes *P* < 0.0001, n.s. denotes not significant.

Next, we examined the expression of cleaved CASP8, the initiator caspase of extrinsic apoptosis, to investigate whether FCL activated extrinsic apoptosis. Indeed, FCL stimulated the expression of cleaved CASP8 in both EC1 and ECA109 cells (**Figure 5A**). To further define the potential mechanism of FCL-induced extrinsic apoptosis, the expression of death receptor family members Fas, DR3, and DR5 were determined. Our results showed that FCL significantly induced the expression of death receptor DR5 both at protein and mRNA levels (**Figures 5B, C** **)**, indicating that DR5 was involved in extrinsic apoptosis upon FCL treatment. To support this notion, the expression of DR5 was downregulated *via* siRNA silencing. We found downregulation of DR5 with siRNA significantly reduced the FCL-induced extrinsic apoptosis, along with a reduction in cleaved PARP expression (**Figures 5D–F**). These results highlighted the key role of DR5 in extrinsic apoptosis triggered by FCL.

Previous studies reported that transcription factor CHOP, a classical downstream target of ATF4, could transactivated DR5 (22–24). Therefore, we determined whether the induction of ATF4 and CHOP expression was responsible for the FCL-induced DR5 expression. Our study showed that FCL induced the obvious up-regulation of ATF4 and CHOP in EC1 and ECA109 cells (**Figure 5G**), along with an increase at the mRNA level of ATF4 (**Figure 5H**). To further examine whether DR5-induced extrinsic apoptosis upon FCL treatment was ATF4 dependent, ATF4 expression was downregulated by siRNA silencing. We found that downregulation of ATF4 significantly rescued the induction of DR5 both at the mRNA (**Figure 5I**) and protein levels (**Figure 5J**), demonstrating the crucial role of ATF4 in the induction of DR5 upon FCL stimulation. As a result, ATF4 siRNA dramatically diminished the expression of cleaved PARP and cleaved CASP8 (**Figure 5J**). Collectively, these results indicated that FCL activated the extrinsic apoptosis *via* ATF4-DR5 axis in ESCC cells.

## Discussion

**Figure 6:**
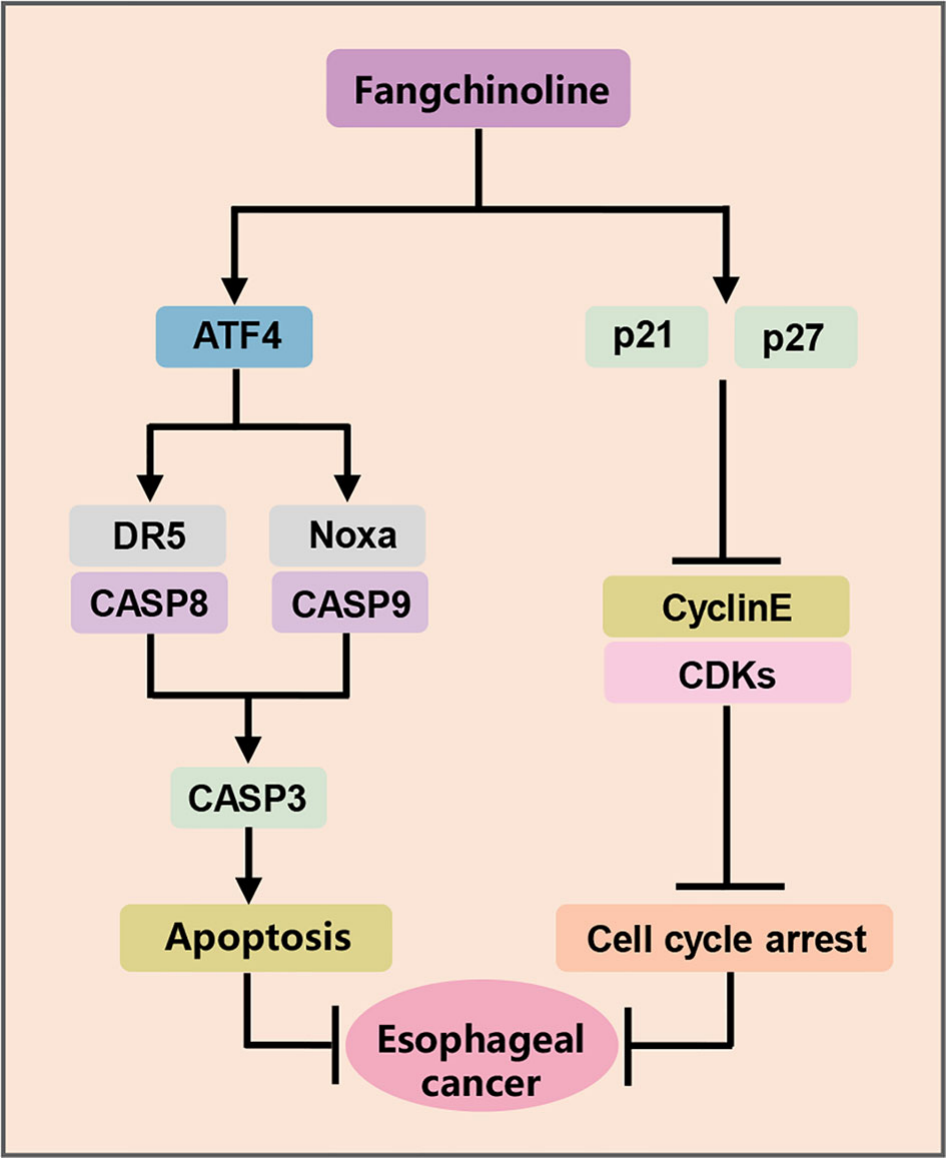
The mechanism of FCL inhibiting the tumor growth of ESCC.

ESCC is one of the most aggressive human malignancies with high incidence and mortality (25). However, few achievements have been achieved in the development of novel anti-ESCC strategies and effective drugs in the past few years (25). Recently, a variety of Chinese herbal extracts and isolated compounds exhibited the substantial anti-tumor efficacy in esophageal cancer cells, and some are candidates for clinical development (26). In the present study, FCL was shown to be a promising anti-ESCC agent with inhibited effects in four ESCC cell lines and in nude mouse xenograft. In mechanisms, FCL-treated ESCC cells arrested in the G1 phase of the cell cycle, which in a p21 and p27-induction manner. Furthermore, FCL transactivated ATF4 to coordinatively trigger Noxa-dependent intrinsic apoptosis and DR5-dependent extrinsic apoptosis (**Figure 6**).

The acceleration of cell cycle process contributes to sustained proliferation and rapid growth of cancer cells. Cyclin dependent kinases (CDKs), such as Cyclin D/E and CDK2/4/6, which are involved in promoting cell cycle progression, are often overexpressed, while cyclin-dependent kinases (CDKIs), such as p21 and p27, are generally downregulated in cancer cells (27). Therefore, suppressing cell cycle progression by controlling cell cycle regulators is considered as an effective strategy to halt tumor growth. FCL was demonstrated to induce G1-S arrest by suppressing the expression of Cyclin D/E and CDK2/4/6 in several human cancers (12, 16, 28). Furthermore, it was reported that FCL restrained the cell cycle progression by inducing the accumulation of p21 and p27 in most malignancies, such as breast cancer cells, prostate carcinoma cancer cells and glioblastoma cells (16, 28, 29). However, the potential role of p21 and p27 in FCL-elicited cell cycle inhibition was unclear. In our study, we found that FCL arrested cell cycle progression at G1 phase by inducing the accumulation of cell cycle inhibitors p21 and p27. Rescue experiments further revealed that additional p21 or p27 knockdown reversed the FCL-induced G1 phase arrest. Therefore, for the first time, we demonstrated that FCL-induced cell-cycle arrest in ESCC is dependent on p21 and p27.

Endoplasmic reticulum (ER) stress, as the common trigger of apoptosis, has been reported to induce CHOP-mediated DR5 transcription and CASP8-mediated extrinsic apoptosis in human cancer cells (30, 31). Previous studies showed that FCL significantly upregulated the ER stress markers including CHOP and ATF4 (32). Therefore, we determined whether FCL activated apoptosis through ATF4-DR5 axis. In this study, we found that, in ESCC cells, FCL induced DR5-mediated extrinsic apoptosis. Moreover, DR5-induced extrinsic apoptosis is ATF4 dependent since downregulation of ATF4 significantly reduced FCL-induced apoptosis. In addition to extrinsic apoptosis, we showed that FCL triggered intrinsic apoptosis in a Noxa-dependent manner. FCL-induced Noxa up-regulation was also ATF4-dependent. However, knockdown of ATF4 did not completely rescue FCL-induced Noxa accumulation (**Figure 4G, H** **)**. Considering several transcription factors (ATF3, p53, NF-κB, and c-Myc, etc) that are known to mediate Noxa gene expression, except for ATF4 (33, 34). The precise regulatory mechanism of Noxa induction elicited by FCL needs further exploration. Furthermore, our study showed that FCL transactivated ATF4 in ESCC cells. It has been reported that some Chinese herbal medicinal agents transactivate ATF4 by inducing ER stress. For example, Zerumbone and Parthenolide activated eIF2α through ER stress, thus inducing the transcription of ATF4 in human colon cancer cells and lung cells (35, 36). Therefore, FCL may also transactivate ATF4 through ER stress. Future studies will be performed to elucidate the mechanism by how FCL transactivates ATF4 in esophageal cancer cells.

In conclusion, our study highlighted a pivotal role of FCL in suppressing the tumor progression of ESCC both *in vitro* and *in vivo*, and discovered a novel mechanism of FCL induction of both intrinsic and extrinsic apoptosis in ESCC, suggesting that FCL was a potential anti-ESCC agent.

## Data Availability Statement

The original contributions presented in the study are included in the article/**Supplementary Material**. Further inquiries can be directed to the corresponding authors.

## Ethics Statement

The animal study was reviewed and approved by Animal Experimental Ethics Committee of Longhua hospital, Shanghai University of Traditional Chinese Medicine.

## Author Contributions 

YJZ, SW, and LJ designed the study. YJZ and SW conducted most of the experiments and wrote the manuscript. YJZ and SW analyzed the data and contributed to the manuscript completion. YC, JZ, JY, JX, and LL offered technical support. HZ and YMZ provided the reagents. RMH, YMZ, and LJ revised the manuscript. All authors contributed to the article and approved the submitted version.

## Funding

This research was funded by the National Natural Science Foundation of China (Grant numbers 81820108022, 81625018, 81602072, 81902380), Innovation Program of Shanghai Municipal Education Commission (Grant number 2019-01-07-00-10-E00056), Program of Shanghai Academic/Technology Research Leader (Grant number 18XD1403800).

## Conflict of Interest

RMH was employed by Anticancer Inc.

The remaining authors declare that the research was conducted in the absence of any commercial or financial relationships that could be construed as a potential conflict of interest.

## Supplementary Material

Supplementary Figure 1 Fangchinoline inhibited ESCC proliferation *in vitro*. **(A)** ESCC cell lines EC1, ECA109, Kyse150 and Kyse450 were treated with indicated concentrations of FCL for 24 hours, and then for photographed under microcopy. Representative images are shown, scale bar = 100μm.
