# Supplementary figures and images for: Fangchinoline Inhibits Human Esophageal Cancer by Transactivating ATF4 to Trigger Both Noxa-Dependent Intrinsic and DR5-Dependent Extrinsic Apoptosis
Source: Front Oncol. 2021 Jun 14;11:666549. doi: 10.3389/fonc.2021.666549 (PMC8236818; doi:10.3389/fonc.2021.666549)

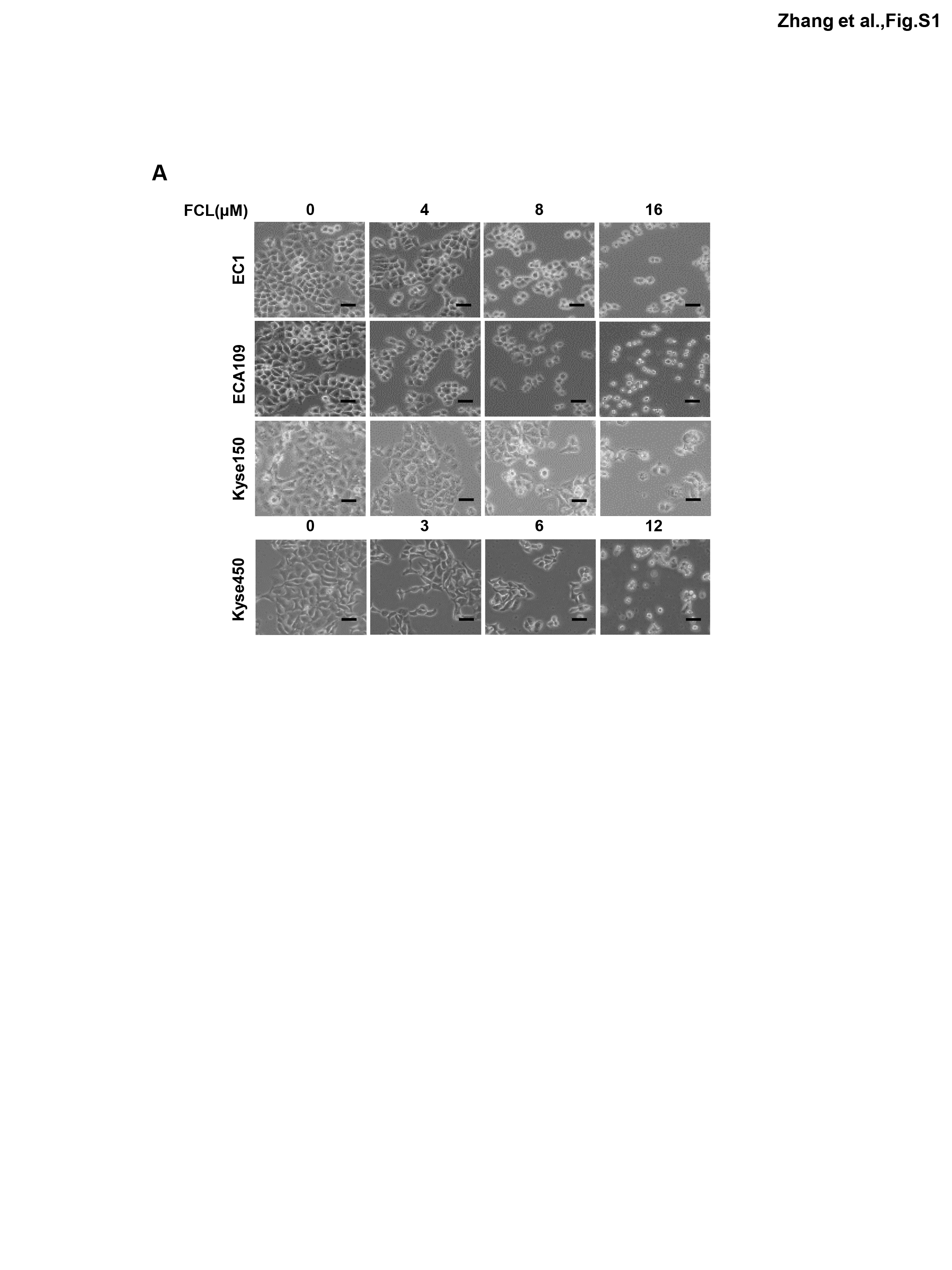

Supplement: Supplementary Figure 1 — Fangchinoline inhibited ESCC proliferation in vitro. (A) ESCC cell lines EC1, ECA109, Kyse150 and Kyse450 were treated with indicated concentrations of FCL for 24 hours, and then for photographed under microcopy. Representative images are shown, scale bar = 100μm. [file Image_1.tif]
